# Investigating the potential selective effects of a Kiperin multi-component magnesium supplement on colon cancer and normal cells

**DOI:** 10.1038/s41598-025-32854-6

**Published:** 2025-12-24

**Authors:** Lutfiye Karcioglu Batur, Cuneyd Yavas, Yalçın Polat

**Affiliations:** 1 Department of Molecular Biology and Genetics, Faculty of Engineering and Natural Sciences, Biruni University, 34015 Istanbul, Türkiye; 2 Biruni University Research Center (B@MER), Biruni University, 75 Sk No:1-13 M. G, 34015 Merkezefendi, Istanbul, Türkiye; 3 Division of Surgical Medical Sciences, Department of Medical Pathology, Faculty of Medicine, Istanbul Atlas University, Istanbul, Türkiye

**Keywords:** Kiperin multi-component magnesium, HCT116 colon cancer, CCD-18Co human colon fibroblasts, Potential selective effects, Cancer, Cell biology

## Abstract

Magnesium is vital for numerous cellular processes and has demonstrated therapeutic potential in various physiological and pathological contexts. Colorectal cancer remains a major cause of cancer-related mortality worldwide. This study evaluates the effects of a multi-component magnesium supplement on human colon fibroblasts (CCD-18Co) and colon cancer cells (HCT-116). CCD-18Co and HCT-116 cells were treated with 2.5 μL dose of the Kiperin multi-component magnesium supplement. Cell viability was assessed via cell counting, and migration was analyzed using wound healing assays. Oxidative stress was evaluated by measuring total oxidant status (TOS) and total antioxidant status (TAS). Gene expression levels of VDR and TNFα genes were measured by Real-Time PCR. Magnesium complex supplementation significantly reduced HCT-116 viability (*p* < 0.001) while enhancing CCD-18Co proliferation (*p* < 0.01). Cell migration increased in CCD-18Co cells at 18 h (*p* < 0.05), while HCT-116 migration decreased at 6 h (*p* < 0.05) and increased at 18 h. TOS levels were significantly decreased in both cell lines, indicating reduced oxidative stress. Additionally, magnesium supplementation upregulated *VDR* expression and downregulated *TNFα* in CCD-18Co cells. Similarly, in HCT-116 colon cancer cells, *VDR* expression was significantly increased, whereas *TNFα* expression showed no statistically significant change. Magnesium supplement may exert selective effects by inhibiting colon cancer cell survival while supporting proliferation and anti-inflammatory signaling in normal colon cells. These findings highlight its potential utility in colon cancer prevention and gut health.

## Introduction

Metal ions are essential during both pathological and physiological processes within living organisms. Magnesium, a primary intracellular divalent cation, is essential for maintaining cellular homeostasis and is involved in nearly all cellular activities^1^. In the human body, it is predominantly found within cells, with approximately 50–65% stored in bones and the remaining portion distributed across muscles, soft tissues, and organs. Less than 2% of total body magnesium exists in extracellular fluids and blood plasma. The homeostasis of magnesium is tightly regulated through intestinal absorption, renal reabsorption/excretion, and its exchange from bone stores. Disruptions in magnesium balance, such as hypomagnesemia or chronic magnesium deficiency, have been linked to various pathological conditions, including metabolic disorders, cardiovascular diseases, and neurological dysfunctions^2,3^. Conversely, hypermagnesemia, though rare, can lead to neuromuscular impairments and cardiovascular complications^4,5^.

Due to its diverse physiological functions, magnesium has garnered significant attention in nutritional and biomedical research. Various forms of magnesium supplements, including citrate, bisglycinate, malate, taurate, and L-threonate, differ in bioavailability and therapeutic applications. Magnesium citrate is highly bioavailable and benefits digestive health^6^, while magnesium glycinate is known for its calming effects on the nervous system^7^. Magnesium malate contributes to energy metabolism and muscle function^8^, whereas magnesium taurate is associated with cardiovascular health^9^. Magnesium L-threonate, uniquely capable of crossing the blood–brain barrier, has demonstrated potential in cognitive enhancement and neuroprotection^9^.

Magnesium plays a crucial role in numerous biological processes relevant to colorectal carcinogenesis, including DNA repair, and inflammation control. Deficiency of magnesium is a major cause of chronic low-grade inflammation, which underlies various pathological conditions such as cardiovascular disease, hypertension, diabetes, and cancer. Furthermore, low magnesium levels have been proposed as a biomarker of poor prognosis, especially in patients receiving agents such as cetuximab or bevacizumab. In addition, magnesium deficiency can impair DNA repair and contribute to genomic instability^10,11^. Complementary animal studies further support a protective role of magnesium: organo-magnesium compounds inhibited inflammation-related colon carcinogenesis in mice by modulating cell proliferation, chromosomal stability, and colonic inflammation, suggesting potential chemopreventive applications in inflammation-driven colorectal cancer^12^.

Globally, colorectal cancer remains among the most common and lethal malignancies, responsible for approximately 1.9 million new cases and over 900,000 deaths in 2022^13^. Maintaining sufficient levels of vitamin D alongside adequate magnesium intake may play a crucial role in reducing mortality risk among colorectal cancer patients^14^. Epidemiological data indicate that higher dietary magnesium intake is associated with a modest reduction in colorectal cancer risk, particularly for colon cancer^15–17^. In the Iowa Women’s Health Study, which followed 35,196 women for 17 years, 1112 colorectal cancer cases were identified. Women with the highest dietary magnesium intake had a significantly lower risk of colon cancer, although no association was observed for rectal cancer^18^. Similarly, magnesium intake around 400 mg/day from both dietary and supplemental sources was linked to a lower incidence of colorectal cancer in postmenopausal women^19^.

The magnesium supplement used in this study (Kiperin) is a multi-component formulation containing magnesium taurate, magnesium bisglycinate, magnesium malate, magnesium citrate, and magnesium L-threonate. While direct evidence for the role of these specific compounds in colon cancer is limited, some components show promising biological activity. Taurine, a constituent of magnesium taurate, has demonstrated antioxidant and anti-inflammatory effects and enhanced the efficacy of 5-FU in a colon cancer model^20^. Magnesium bisglycinate, known for its high bioavailability, has been associated with improved cisplatin-mediated tumor suppression and reduced nephrotoxicity^21^. Although magnesium malate lacks direct evidence in colon cancer, its inclusion reflects magnesium’s general involvement in cellular function and tumor response^21^. Magnesium citrate primarily aids bowel preparation for colonoscopy without direct anti-cancer activity^22–24^, while magnesium L-threonate may support symptom control in advanced cancer patients, particularly in pain management, though its role in tumor biology remains unclear^25^.

Given the critical roles of magnesium in cellular homeostasis and disease prevention, the present study aims to evaluate the antioxidant, cytotoxic, and genotoxic effects of a multi-component magnesium supplement containing magnesium taurate, bisglycinate, malate, citrate, and L-threonate. The study investigates the effects of the magnesium supplement on healthy human colon fibroblast cells (CCD-18Co) and human colon cancer cells (HCT-116). By analyzing key cellular mechanisms, we aim to provide valuable insights into how magnesium affects cellular behavior at the molecular level, potentially contributing to a better understanding of magnesium’s role in health and disease.

## Materials and methods

### Cell culture

This study was approved by the Scientific Research Ethics Committee of Biruni University (Decree No: 2024-BIAEK/06–47, dated January 20, 2025). Two cell lines were used: CCD-18Co human colon fibroblast cells (CRL-1459) and HCT-116 human colon cancer cells (CCL-247), both obtained from the American Type Culture Collection (ATCC). CCD-18Co cells was used as a control cell line. Cells were cultured in High Glucose Dulbecco’s Modified Eagle Medium (HG-DMEM) supplemented with 10% fetal bovine serum (FBS) and 1% penicillin–streptomycin, and maintained at 37 °C in a humidified incubator with 5% CO_2_ throughout the experimental procedures.

A commercially available multi-component magnesium supplement was used as the test compound. This product, obtained from Kiperin Pharmaceutical Food Inc. (Istanbul, Turkey), contained magnesium taurate, magnesium bisglycinate, magnesium malate, magnesium citrate, and magnesium L-threonate. A total of 8 mg of magnesium was dissolved in 40 mL of phosphate-buffered saline (PBS) (main stock 200 ppm), and six different concentrations (0.5 µL, 1 µL, 1.5 µL, 2 µL, 2.5 µL, and 3 µL) were tested.

For dose optimization, CCD-18Co cells were seeded into six-well plates and incubated for 24 h. Once the cells reached approximately 70% confluence, wound healing (scratch) assays were performed, followed by treatment with the different magnesium concentrations. Initial imaging was performed using the ZOE Fluorescent Cell Imager**.** Based on the results, the 2.5 µL (1.278 µM; 0.25 ppm**)** concentration was identified as the most effective and was used for all subsequent analyses. Non-treated cells group was used as a control group for both cell lines.

### Wound healing assay

To evaluate cell migration in response to magnesium treatment, a wound healing (scratch) assay was performed using a concentration of 2.5 µL of the magnesium supplement. Confluent monolayers (≥ 80%) of CCD-18Co and HCT-116 cells were scratched using a sterile 200 µL pipette tip to create a uniform wound. Images of the wound area were captured at 0-, 6-, 18-, and 24-h post-scratch using the ZOE Fluorescent Cell Imager, consistently positioned at the same marked field of view for each time point. Cell migration was quantified by measuring the percentage of wound closure over time.

### Measurement of oxidative stress levels and antioxidant capacity

Cells were treated with the magnesium supplement for 24 h prior to measurement of oxidative stress and antioxidant capacity. Cell lysates were prepared from CCD-18Co and HCT-116 cell lines according to standard protocols. The resulting supernatants were used for the quantification of total oxidant status (TOS) and total antioxidant status (TAS), while cell pellets were reserved for RNA isolation. TOS and TAS levels were measured using commercial assay kits provided by Rel Assay Diagnostics (Mega Tıp Inc., Gaziantep, Turkey), in accordance with the manufacturer’s instructions. Spectrophotometric analysis was performed to assess the oxidative and antioxidative properties of the samples.

For the measurement of TOS, the assay relied on the principle that oxidants in the sample convert ferrous ions (Fe^2+^) into ferric ions (Fe^3+^). These ferric ions then react with a chromogenic agent in an acidic environment to form a colored complex, the intensity of which reflects the oxidant concentration. In the procedure, 45 µL of each sample (cell lysate, standard, or blank/distilled water) was combined with 300 µL of buffer solution (pH 1.75). Absorbance was measured at 530 nm after 30 s. Next, 15 µL of ferrous ion reagent was added, and the mixture was incubated for 5 min at 37°C (or 10 min at room temperature), followed by a second absorbance reading. The TOS levels were calculated and reported as micromolar hydrogen peroxide equivalents per liter (µmol H_2_O_2_ Equiv./L).

For TAS analysis, the method is based on the ability of antioxidants present in the sample to neutralize ABTS radical cations, which are initially dark blue-green in color. Upon reduction, these radicals become colorless, and the degree of decolorization corresponds to the antioxidant capacity. To perform the assay, 18 µL of each sample (cell lysate, standard solution, or distilled water/blank) was mixed with 300 µL of acetate buffer (pH 5.8). The absorbance was first measured at 660 nm after 30 s. Then, 45 µL of ABTS prochromogen solution was added, and following incubation for 5 min at 37°C (or 10 min at room temperature), the final absorbance was recorded. TAS levels were calculated and expressed as millimoles of Trolox equivalents per liter (mmol Trolox Eq/L).

Both TOS and TAS values were statistically evaluated and are reported as mean ± standard deviation (SD).

### Quantitative real-time PCR for *VDR *and *TNFα* genes expression

Total RNA was extracted from cells, and RNA concentration was determined using Nanodrop for CCD-18Co (500 ng threshold) and Qubit for HCT-116 (400 ng threshold). The same RNA isolation kit was used for both cell lines. Subsequently, 500 ng of RNA was reverse transcribed into complementary DNA (cDNA) using the OneScript Plus cDNA Synthesis Kit (Applied Biological Materials Inc., Richmond, Canada) according to the manufacturer’s protocol. cDNA synthesis was performed in a thermal cycler (T100 Thermal Cycler, Bio-Rad, Singapore) at 52°C for 15 min.

Quantitative real-time PCR (qPCR) was performed using BlasTaq 2X qPCR MasterMix (Applied Biological Materials Inc.) under the cycling conditions shown in Table 1. Each reaction contained 2 µL of cDNA template, 0.5 µL of each primer (10 µM), and 10 µL of Master Mix, with a final volume adjusted to 20 µL using nuclease-free water. The forward and reverse primer sequences used in the study are listed in Table 2. Fluorescence data were collected at the end of each elongation step, and gene expressions were analyzed using the 2^−ΔΔCt^ method, with GAPDH serving as the internal control.

**Table 1:** The qPCR cycles protocol for BlasTaq™ 2X qPCR MasterMix.

Cycle step		Temperature (°C)	Time	Cycles
Enzyme activation		95	3 min	1
Quantification	Denaturation	95	15 s	45
	Annealing	60	15 s	
	Elongation	72	15 s	
Cooling		40	20 s	1

**Table 2:** Forward and reverse primer sequences of the studied genes.

Gene	Forward primer	Reverse primer
*VDR*	5′-CCAAGCTGTCTGAGGAGCAG-3′	5′-GAACTGGAGGCCGGAACTG-3′
*TNFα*	5′-CAGCCTCTTCTCCTTCCTGAT-3′	5′-GCCAGAGGGCTGATTAGAGA-3′
*GAPDH*	5′-CCACCCATGGCAAATTCC-3′	5′-TGGGATTTCCATTGATGACAAG-3′

### Statistical analysis

All statistical analyses were performed using GraphPad Prism software (version 10.4.1). Data are presented as mean ± standard deviation (SD). For comparisons involving more than two groups, ANOVA was applied to assess overall statistical significance. Two-way analysis of variance (two-way ANOVA) was used to evaluate the effects of treatment, time, and their interaction on wound closure. When ANOVA indicated significant differences, Tukey’s post hoc test was conducted to determine pairwise group differences. For comparisons between two independent groups, independent samples t-test was employed. A *p*-value of less than 0.05 was considered statistically significant (*p < 0.05, **p < 0.01, *p < 0.001, ns: not significant).

## Results

### Cell viability

**Fig. 1:**
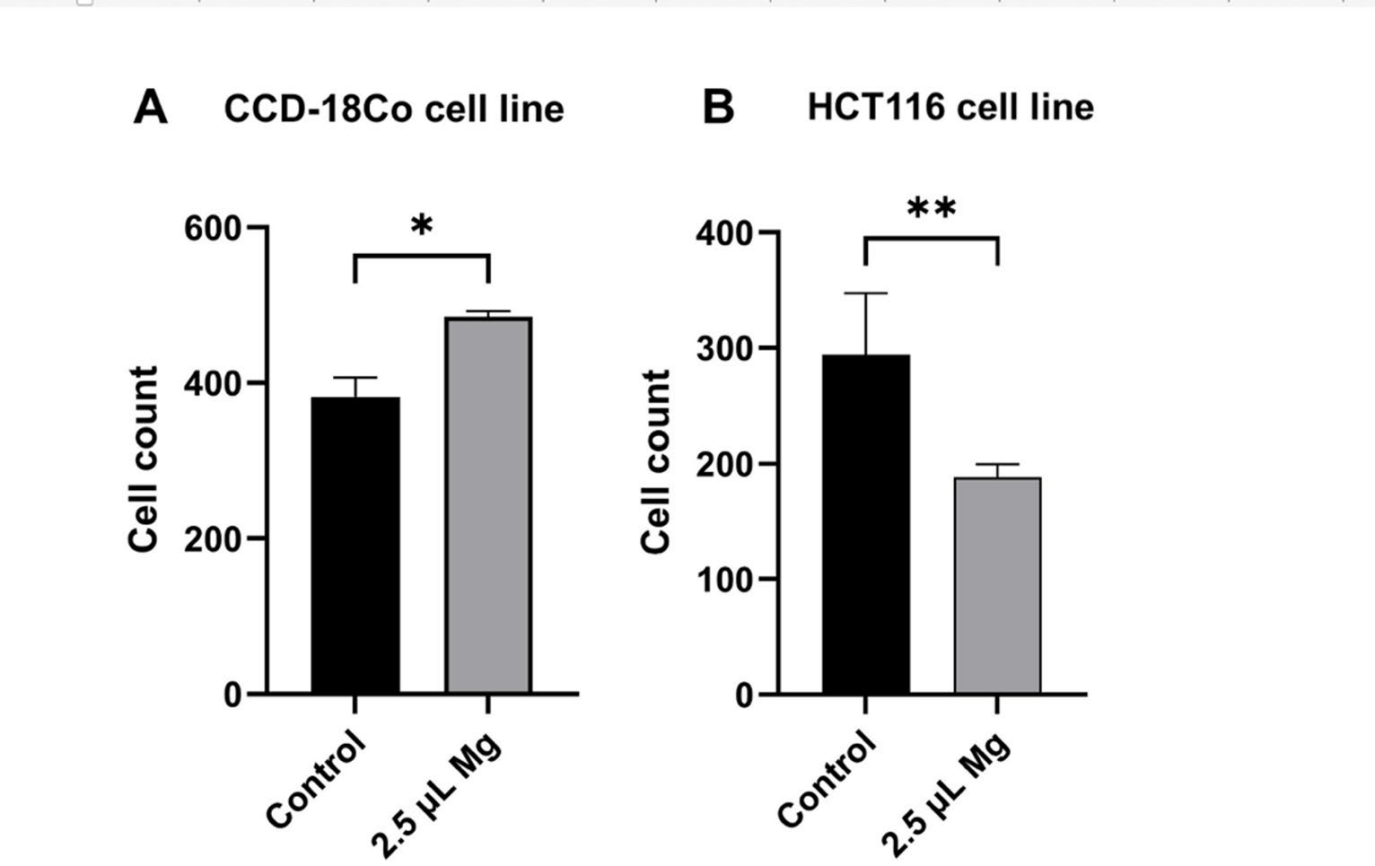
Cell counts of HCT-116 and CCD-18Co cell lines following treatment with 2.5 μL of magnesium. Non-treated cells group was used as a control group. The treatment led to a decrease in the number of HCT-116 cells, whereas an increase was observed in CCD-18Co cells.

The cell numbers of HCT116 and CCD-18Co cell lines were assessed under the control conditions and after treatment with 2.5 µL dose of magnesium complex. The results indicate a significant decrease in cell number of HCT116 cells treated with magnesium compared to the control group (*p* < 0.001). Conversely, the cells numbers of CCD-18Co cell lines were significantly increased in the cells treated with magnesium complex compared to the control group (*p* < 0.01). These findings suggest that treatment with Kiperin multi-component magnesium supplement has inhibitory effects on the cell growth of colon cancer cells, but it increases the growth of normal colon cells (Fig. 1).

### Assessment of cell migration using the wound healing (scratch) assay

**Fig. 2:**
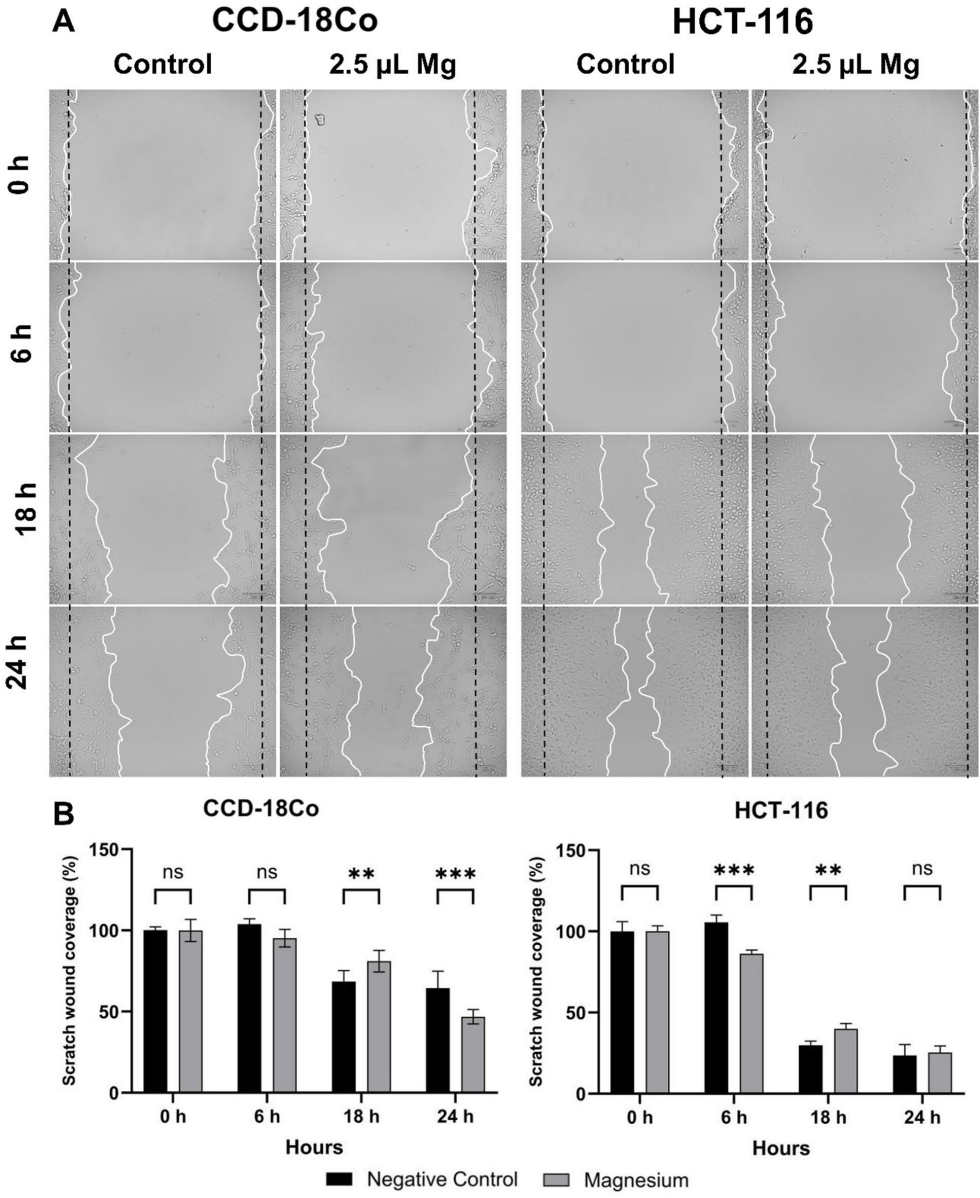
Scratch wound assay for evaluating the cell migration of CCD-18Co and HCT116 cell lines at 0, 6, 18 and 24 h after 2.5 μL of magnesium treatment. Non-treated cells group was used as a control group.

According to the wound healing assay results, treatment with 2.5 μL of Kiperin multi-component magnesium supplement significantly increased the migration of CCD-18Co cells compared to control cells at 18 h (*p* < 0.05). In contrast, in the HCT-116 cell line, magnesium supplement at the same dose significantly reduced cell migration at 6 h (*p* < 0.05) but led to an increase in migration at 18 h (Fig. 2).

### Total oxidative and antioxidant status

The TOS levels in HCT-116 cells significantly decreased following treatment with 2.5 μL magnesium supplement compared to the control group (3.68 ± 1.19 vs. 4.34 ± 0.58 µmol H_2_O_2_ equiv/L, *p* = 0.032). This suggests a reduction in oxidative stress associated with magnesium administration. Although the TAS levels in the same group showed an increase (0.86 ± 0.47 vs. 0.09 ± 0.04 mmol Trolox equiv/L), this difference did not reach statistical significance (*p* = 0.095). Nonetheless, the marked elevation in antioxidant capacity may imply a potential modulatory effect of magnesium supplement on redox homeostasis in HCT-116 cells (Table 3).

**Table 3:** Total oxidative status (TOS) and total antioxidant status (TAS) levels in CCD-18Co and HCT-116 groups treated with 2.5 μL of magnesium supplement.

HCT116 Cells	TOS (µmol H_2_O_2_ Equiv/L)	TAS (mmol Trolox Equiv/L)
Control	4.34 ± 0.58	0.09 ± 0.04
H_2_O_2_	6.76 ± 1.75	0.25 ± 0.08
2.5 μL Magnesium	3.68 ± 1.19^a^	0.86 ± 0.47^b^
*P* value	**0.020**	**0.004**
CCD-18Co Cells	TOS (µmol H_2_O_2_ Equiv/L)	TAS (mmol Trolox Equiv/L)
Control	14.85 ± 1.57	0.64 ± 0.16
2.5 μL Magnesium	10.19 ± 1.62^b^	0.45 ± 0.29
*P* value	**0.019**	0.510

TOS: Total oxidant status, TAS: Total antioxidant status.

All values are presented as mean ± standard deviation. ^a^*p* < 0.05 vs. H_2_O_2_ group; ^b^*p* < 0.05 vs. control group. Bold *p* values are significant.

**Fig. 3:**
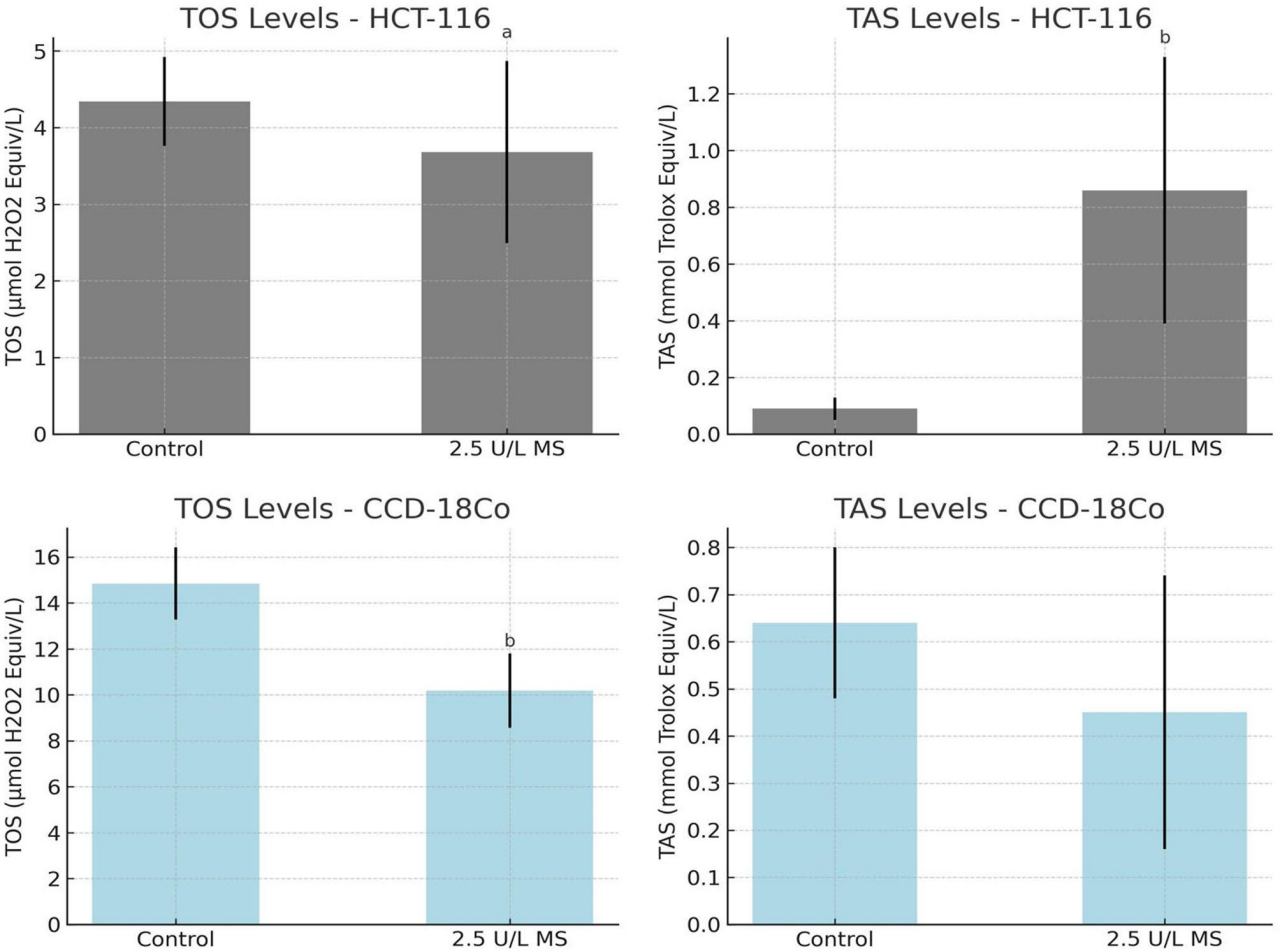
Total oxidative status (TOS) and total antioxidant status (TAS) levels in CCD-18Co and HCT-116 groups treated with 2.5 μL of magnesium supplement.

As shown in Fig. 3, CCD-18Co cells exposure to 2.5 μL magnesium supplement led to a statistically significant reduction in TOS levels compared to the control group (10.19 ± 1.62 vs. 14.85 ± 1.57 µmol H_2_O_2_ equiv/L, *p* = 0.019), indicating a decrease in oxidative stress. TAS levels, however, exhibited a slight decline in the magnesium treated group (0.45 ± 0.29 vs. 0.64 ± 0.16 mmol Trolox equiv/L), but this change was not statistically significant (*p* = 0.510) (Table 3).

### Expression levels of *VDR* and* TNFα* genes

**Fig. 4:**
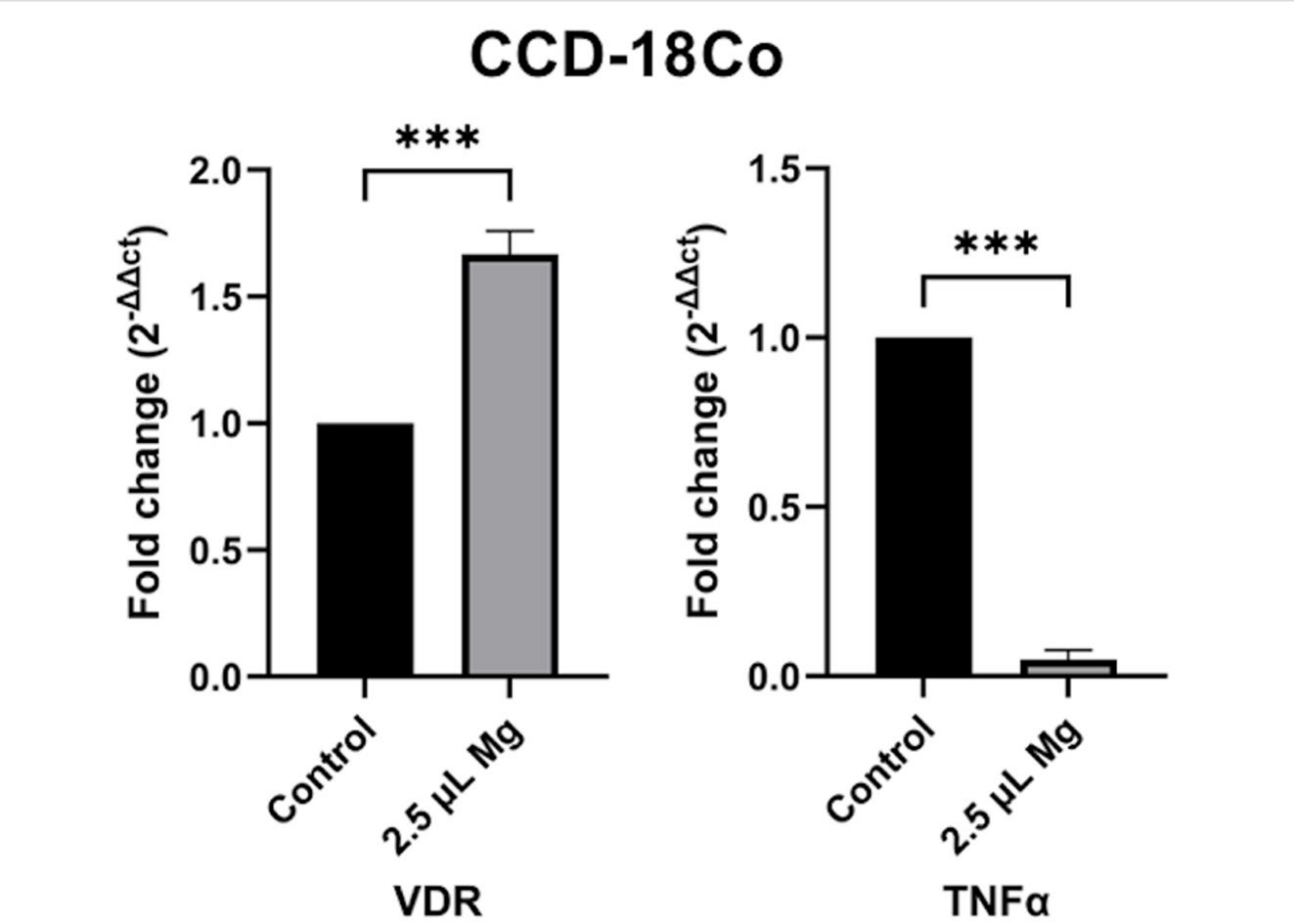
Gene expression in CCD-18Co cells with and without 2.5 μL magnesium supplement. *VDR* and *TNFα* gene expression levels were significantly altered. Non-treated cells group was used as a control group.

Following treatment with 2.5 μL of Kiperin multi-component magnesium supplement, the expression levels of the *VDR* and *TNFα* genes were assessed in both HCT116 and CCD-18Co cell lines. In CCD-18Co cells, magnesium supplementation significantly upregulated *VDR* expression and downregulated *TNFα* expression, as shown in Fig. 4 (*p* < 0.0001). These findings suggest that magnesium promotes anti-inflammatory and regulatory gene signaling in normal colon fibroblasts.

**Fig. 5:**
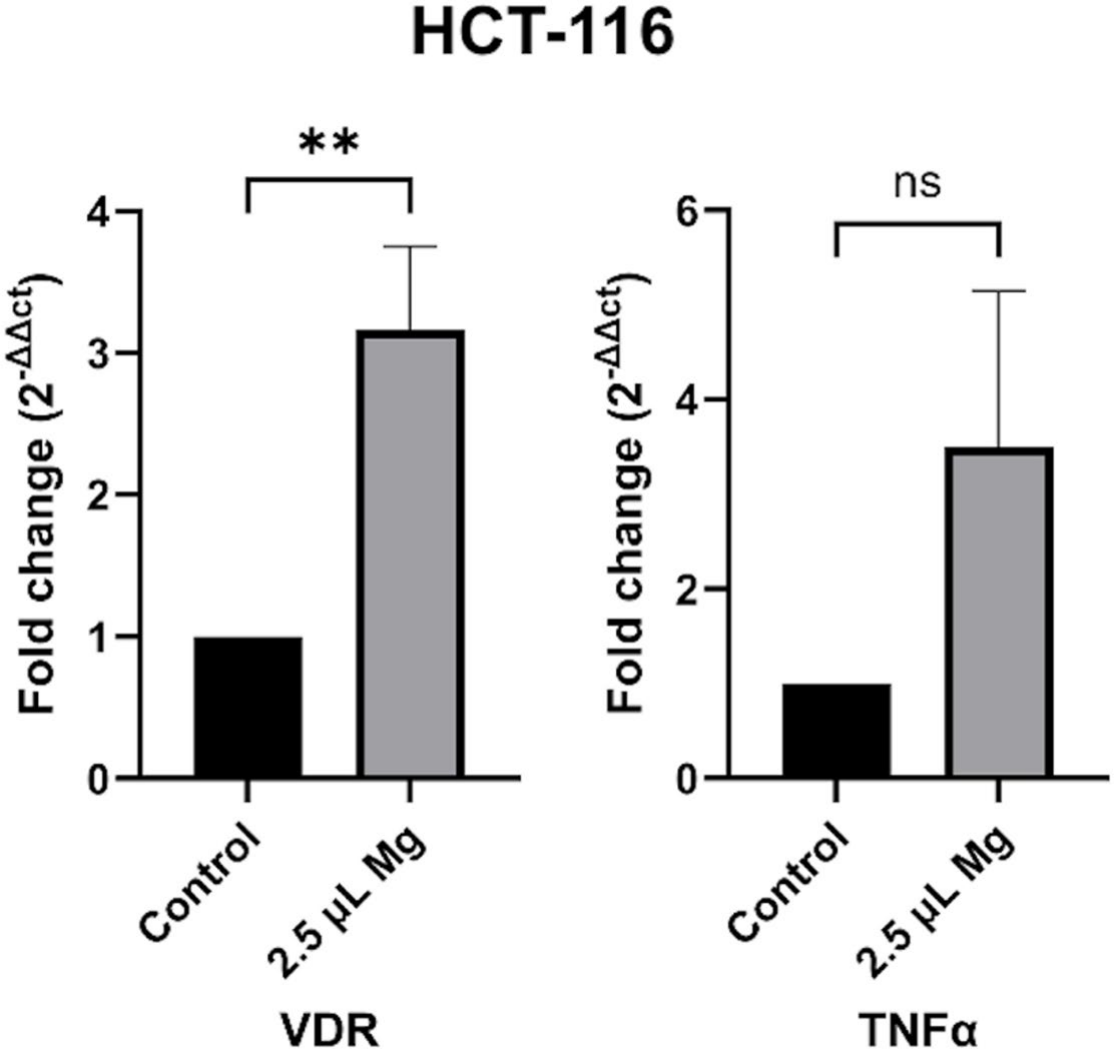
Gene expression in HCT-116 cells with and without 2.5 μL magnesium supplement. *VDR* gene expression levels were significantly increased. Non-treated cells group was used as a control group.

Moreover, in colon cancer cell line HCT-116, *VDR* gene expression levels also increased following magnesium supplement; however, no statistically significant change was observed in *TNFα* expression, as shown in Fig. 5.

## Discussion

Magnesium, the second most abundant intracellular cation, plays a critical role in enzyme regulation, energy metabolism, cell proliferation, and neuromuscular function^26^. Magnesium deficiency has been associated with increased tumor development, while supplementation shows promise in slowing tumor progression. Its use in anti-tumor implants, which release magnesium ions and other bioactive byproducts, has demonstrated anti-inflammatory and antioxidant effects, further supporting its therapeutic potential^27,28^. A growing body of evidence links magnesium status with colorectal cancer development and progression^29,30^, pointing to the potential for magnesium supplementation as a preventive and therapeutic tool.

Despite encouraging findings, the integration of magnesium into standard cancer therapies remains limited. Therefore, this study aimed to evaluate the effects of a multi-component magnesium supplement on colon cancer (HCT-116) and normal colon fibroblast (CCD-18Co) cells in vitro, focusing on cell viability, migration, oxidative stress, and gene expression related to inflammation and vitamin D signaling. Our findings demonstrate that magnesium treatment significantly reduced HCT-116 colon cancer cell numbers while increasing CCD-18Co fibroblast proliferation. Furthermore, magnesium influenced cell migration in a time- and cell-type-dependent manner. It enhanced migration of CCD-18Co fibroblasts at 18 h but initially reduced HCT-116 migration at 6 h, followed by an increase at 18 h. These biphasic and differential effects may reflect distinct regulatory mechanisms between normal and cancerous cells, the initial reduction in migration at 6 h may reflect an acute cytostatic or stress-adaptive phase following magnesium exposure, during which transient changes in intracellular ion balance and ATP-dependent signaling can temporarily suppress motility. The subsequent increase in migration at 18 h is likely a compensatory response associated with magnesium’s regulatory role in cytoskeletal remodeling, energy metabolism, and focal adhesion signaling. Comparable time-dependent dual effects of magnesium on proliferation and migration have been reported in different cell types, supporting this interpretation^15,26,31^. These results aligning with the findings of Wesselink et al.^14^, who reported significantly lower mortality among colorectal cancer patients with adequate vitamin D and magnesium levels. Supporting its clinical relevance, previous studies have shown beneficial effects of magnesium, including reduced hypercoagulability during colon cancer surgery^32^. Additionally, in a murine model of inflammation-associated colorectal cancer, magnesium supplementation suppressed tumor formation^12^. Meta-analyses also confirm that higher dietary magnesium intake is modestly, but consistently associated with reduced colorectal cancer risk^30^.

Magnesium is also critical for mitochondrial stability and redox balance. In deficiency states, mitochondrial magnesium levels decrease, leading to impaired electron transport, increased oxidant generation, and reduced activity of antioxidant enzymes^33–35^. In our study, magnesium supplementation significantly lowered total oxidant status in both colon cancer and normal fibroblast cells, reinforcing its antioxidant role. Magnesium also counteracts calcium-mediated oxidant production by inhibiting calcium-dependent enzymes such as nitric oxide synthase^36^.

In cancer studies, it has been reported that drugs and drug candidates characterised as anticancer drugs cause increases or decreases in different time periods^37,38^. This phenomenon may indicate a time-dependent cellular adaptation to the bioactive compounds present in the supplement^39^. The early inhibition of migration could be attributed to a transient cytostatic or stress-induced response, possibly linked to redox imbalance or modulation of cytoskeletal dynamics. However, despite the late adaptive increase in motility, the overall proliferative capacity of cancer cells remained suppressed compared to normal cells, suggesting that the supplement selectively limits malignant cell growth while allowing healthy cells to maintain their physiological behavior. In our study, the wound healing assay in HCT-116 cells demonstrated a biphasic response, characterized by an initial reduction in cell migration at 6 h followed by a subsequent increase at 18 h.

Moreover, magnesium suppresses inflammatory signaling cascades by inhibiting *NF-κB* activation and reducing the expression of pro-inflammatory cytokines, including *IL-6* and *TNFα*^40^. These anti-inflammatory effects are well-supported in the literature, including findings that magnesium sulfate protects neurons by reducing oxidant stress and inflammation^41^. Expression of *VDR* is high in normal intestinal epithelium and plays a key role in mediating the anti-cancer effects of vitamin D^42^. However, colon cancer cells, including HCT-116, often exhibit reduced *VDR* levels, which are associated with worse prognosis^43,44^. In our study, magnesium increased *VDR* expression in both normal colon fibroblasts and HCT-116 colon cancer cells. Furthermore, *TNFα* is a central cytokine in cancer-related inflammation and correlates with tumor stage and lymph node involvement in colon and colorectal cancer^45,46^. It is also implicated in other chronic inflammatory conditions such as psoriasis and lupus^47^. Guo et al. demonstrated that *TNFα* promotes immune evasion in colorectal cancer through regulatory T cell recruitment via chemokine receptor 8^48^. It also stimulates secretion of *IL-6* and *IL-8*, promoting tumor-supportive inflammation^49^. In our study, magnesium treatment significantly reduced *TNFα* expression in normal fibroblasts, supporting its anti-inflammatory effect.

Taken together, our findings demonstrate that magnesium supplementation selectively inhibits colon cancer cell viability while promoting proliferation, antioxidant defense, and anti-inflammatory signaling in normal colon fibroblasts. These dual actions support magnesium’s potential as both a therapeutic and preventive agent in colon cancer.

In summary, multi-component magnesium supplement inhibited proliferation in HCT-116 cancer cells while promoting growth and migration in CCD-18Co fibroblasts. It also reduced total oxidant status in both lines, indicating decreased oxidative stress. In normal colon fibroblasts, *VDR* was upregulated and *TNFα* downregulated, suggesting anti-inflammatory and regenerative effects. In cancer cells, *VDR* expression increased significantly, while *TNFα* remained unchanged. These findings highlight magnesium’s potential to support colon health and possibly inhibit tumor progression. Further studies are needed to evaluate its clinical applicability.

## Conclusion

This study provides preliminary evidence that The Kiperin multi-component magnesium supplementation may exert selective regulatory effects on colon cell physiology, promoting cellular balance and antioxidant defense while potentially limiting tumor-promoting mechanisms. These results point to magnesium’s broader role in maintaining colonic homeostasis and modulating cancer-related pathways. Future in-depth studies are essential to confirm these observations and to determine whether magnesium supplementation could be integrated into preventive or supportive strategies for colon cancer management.

## Data Availability

The qPCR data supporting the findings of this study are available from the corresponding author upon reasonable request. No high-throughput sequencing or large-scale gene expression data were generated in this study.
